# Diethyl 4,4′-(ethane-1,2-diyldi­oxy)dibenzoate

**DOI:** 10.1107/S1600536811021258

**Published:** 2011-06-11

**Authors:** Zhen Ma, Huang Yang

**Affiliations:** a School of Chemistry and Chemical Engineering, Guangxi University, Guangxi 530004, People’s Republic of China

## Abstract

The title compound, C_20_H_22_O_6_, was obtained by the reaction of ethyl 4-hy­droxy­benzoate with 1,2-dichloro­ethane in dimethyl­formamide. The mol­ecule lies around the crystallographic inversion center at (0,0,0), with the asymmetric unit consisting of one half of the mol­ecule. The two ethyl groups are in *trans* positions. The ethyl, carboxyl, aryl and O—CH_2_ groups are coplanar with an r.m.s. deviation of 0.0208 (9) Å. The whole mol­ecule is planar with an r.m.s. deviation of 0.0238 (9) Å for the 19 atoms used in the calculation and 0.0071 (9) Å for the two aryl groups in the mol­ecule. A weak inter­molecular C—H⋯O hydrogen bond and a C—H⋯π inter­action help to consolidate the three-dimensional network.

## Related literature

For the synthesis and structures of diesters, see Hou & Kan (2007 ▶); Tashiro *et al.* (1990 ▶); Zhang *et al.* (2007 ▶). For the properties and applications of diesters, see: Chen & Liu (2002 ▶). For the synthesis of the title compound, see: Ma & Liu (2002 ▶); Ma & Cao (2011 ▶). For standard bond lengths, see: Allen *et al.* (1987 ▶).
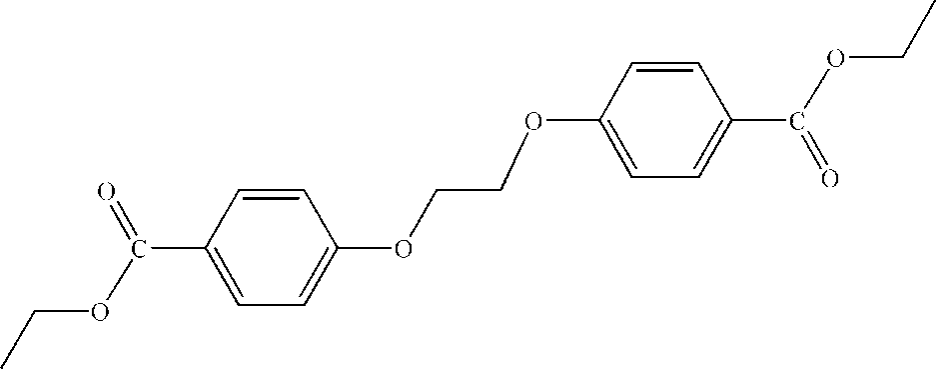


## Experimental

### 

#### Crystal data

C_20_H_22_O_6_
*M* *_r_* = 358.38
Monoclinic, 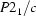

*a* = 4.8504 (10) Å
*b* = 15.847 (3) Å
*c* = 12.0159 (19) Å
β = 104.250 (8)°
*V* = 895.2 (3) Å^3^
*Z* = 2
Mo *K*α radiation
μ = 0.10 mm^−1^
*T* = 298 K
0.49 × 0.35 × 0.22 mm

#### Data collection

Bruker SMART CCD area-detector diffractometer
Absorption correction: multi-scan (*SADABS*; Sheldrick, 1996 ▶) *T*_min_ = 0.960, *T*_max_ = 0.979
8592 measured reflections
1980 independent reflections
1713 reflections with *I* > 2σ(*I*)
*R*_int_ = 0.032

#### Refinement

*R*[*F* ^2^ > 2σ(*F* ^2^)] = 0.039
*wR*(*F* ^2^) = 0.130
*S* = 1.02
1980 reflections
119 parameters
H-atom parameters constrained
Δρ_max_ = 0.34 e Å^−3^
Δρ_min_ = −0.34 e Å^−3^

### 

Data collection: *SMART* (Bruker, 2001 ▶); cell refinement: *SAINT* (Bruker, 2002 ▶); data reduction: *SAINT*; program(s) used to solve structure: *SHELXS97* (Sheldrick, 2008 ▶); program(s) used to refine structure: *SHELXL97* (Sheldrick, 2008 ▶); molecular graphics: *SHELXTL* (Sheldrick, 2008 ▶); software used to prepare material for publication: *SHELXTL*.

## Supplementary Material

Crystal structure: contains datablock(s) I, global. DOI: 10.1107/S1600536811021258/ez2245sup1.cif

Structure factors: contains datablock(s) I. DOI: 10.1107/S1600536811021258/ez2245Isup2.hkl

Supplementary material file. DOI: 10.1107/S1600536811021258/ez2245Isup3.cml

Additional supplementary materials:  crystallographic information; 3D view; checkCIF report

## Figures and Tables

**Table 1:** Hydrogen-bond geometry (Å, °) *Cg* is the centroid of the C4–C9 ring.

*D*—H⋯*A*	*D*—H	H⋯*A*	*D*⋯*A*	*D*—H⋯*A*
C6—H6*A*⋯O2^i^	0.93	2.47	3.2784 (16)	146
C10—H10*B*⋯*Cg*^ii^	0.97	2.65	3.741 (2)	143

Symmetry codes: (i) 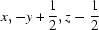; (ii) 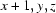.

**Fig. 1.**
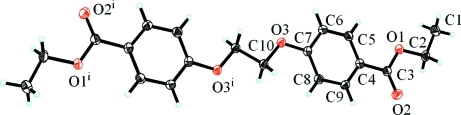
The molecular structure of the title compound with the atom numbering scheme. Displacement ellipsoids are drawn at the 50% probability level. H atoms are presented as small spheres of arbitrary radius. [Symmetry code: (i) -x, -y, -z]

**Fig. 2.**
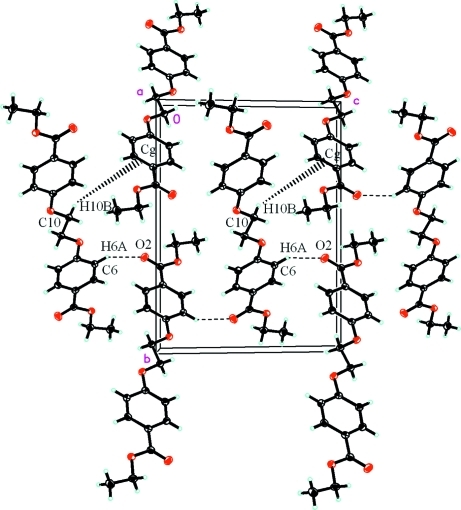
A view of the crystal packing along the a axis. The thin dashed lines are used to show the hydrogen bonds. The thick dashed line is used to show the intermolecular CH-π interactions of –CH2-(C6) and the neighboring aryl groups, from their H atoms to the centroids of the rings of the aryl groups.

**Fig. 3.**
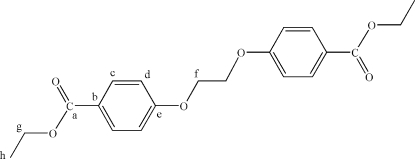
An additional scheme with the numbering scheme used for the NMR spectra.

## supplementary crystallographic information

### Comment

There has been, in recent years, a considerable interest in the study of esters (Hou & Kan, 2007; Tashiro *et al.*, 1990; Zhang *et al.*, 2007), since these compounds are commodity chemicals used as intermediates in the manufacture of acids and to produce many important industrial products. Hence, our current work aims to prepare esters to produce acids and investigate their coordination behaviors with metal ions and study their applications in many fields (Chen & Liu, 2002). Herein, we report a new diester which was obtained by reaction of ethyl 4-hydroxybenzoate with 1,2-dichloroethane in DMF and its structure was confirmed by elemental analysis, IR, NMR spectra and X-ray crystal analysis.

The structure consists of a neutral molecular unit (Fig. 1).The molecule lies on a crystallographic inversion center at (0, 0, 0), thus leading to one half of the molecule being present per asymmetric unit. All bond lengths and angles are within normal ranges (Allen *et al.*, 1987). The ethyl, aryl, carboxyl and the O—CH_2_ groups of one half molecule are coplanar to form one plane with an r.m.s. deviation of 0.0208 (9) Å. By symmetry, the whole molecule is coplanar with an r.m.s deviation of 0.0238 (9) Å for 19 atoms being used for calculation and 0.0071 (9) Å for the two aryl groups at the molecule. Because of the symmetry of the inversion center, the two ethyl groups at the molecule are in a *trans* position. One weak hydrogen bond between one hydrogen atom and the oxygen atom of a neighboring molecule is present in the structure: H6A on C6 and O2^ii^ [symmetry code: (ii) *x*, -*y*+1/2, *z*-1/2] (Table 1). The molecules display intermolecular C—H···π interactions between a –CH_2_-(C10) and a neighboring aryl group [H..Cg 2.647 Å, Cg is the centroid of the six membered ring of C4^iii^-C9^iii^, symmetry code: (iii) *x*+1, *y*, *z*].

### Experimental

The title compound was obtained by the reaction of ethyl 4-hydroxybenzoate with 1,2-dichloroethane in *N*,*N*'-dimethylformamide (DMF) according to a reported procedure (Ma & Liu, 2002; Ma & Cao, 2011). In a 100 cm^3^ flask fitted with a funnel, ethyl 4-hydroxybenzoate (8.3 g, 50 mM) and potassium carbonate were mixed in 50 cm^3^ of DMF. To this solution was added dropwise a stoichiometric quantity of 1,2-dichloroethane (2.5 g, 25 mM) dissolved in 20 cm^3^ of DMF for a period of an hour with stirring. The mixture was then stirred for 24 h at 353 K. The solution was concentrated under reduced pressure and the white solid formed by adding a large quantity of water (200 cm^3^) was filtered off and recrystallized from ethanol and decolored with activated carbon. A colorless solid was obtained (yield 30 %, m.p: 388–390 K). Slow evaporation of a solution of the title compound in ethanol and dichloromethane (1:1) led to the formation of colorless crystals, which were suitable for X-ray characterization. Anal. Calcd. for [C_20_H_22_O_6_] (%): C, 67.03; H, 6.19; found: C, 66.75; H, 6.46; IR(KBr), (cm^-1^): 1711, (C=O), 1605, 1509, 1477 (C=C of aryl), 1280, 1252, 1165, 1105 (CH_2_—O—CH_2_), 1045, 1027, 870-715, (Ar—H). ^1^H NMR (CDCl_3_): 7.97 (d, 4H, *J* = 8.8 Hz, aryl, c), 6.94 (d, 4H, *J* = 8.8 Hz, aryl, d), 4.34 (d, 4H, OCH_2_CH_2_, f), 4.31 (d, 4H, COOCH_2_, g), 1.35 (t, 6H, –CH_3_, h). ^13^C NMR: 166.4 (–COO, a), 162.4 (aryl, b), 131.8 (aryl, c), 123.7 (aryl, e), 114.4 (aryl, d), 66.6 (CH_2_CH_2_, f), 60.9 (CH_2_CH_2_, g), 14.6 (–CH_3_, h). (see Figure 3 for the NMR atom number assignment).

### Refinement

All H atoms were positioned geometrically and refined using a riding model with C—H = 0.93 - 0.97 Å and with *U*_iso_(H) = 1.2 times *U*_eq_(C).

### Crystal data

**Table:**

C_20_H_22_O_6_	*F*(000) = 380
*M**_r_* = 358.38	*D*_x_ = 1.330 Mg m^−^^3^
Monoclinic, *P*2_1_/*c*	Mo *K*α radiation, λ = 0.71073 Å
Hall symbol: -P 2ybc	Cell parameters from 8592 reflections
*a* = 4.8504 (10) Å	θ = 2.2–27.2°
*b* = 15.847 (3) Å	µ = 0.10 mm^−^^1^
*c* = 12.0159 (19) Å	*T* = 298 K
β = 104.250 (8)°	Prism, colorless
*V* = 895.2 (3) Å^3^	0.49 × 0.35 × 0.22 mm
*Z* = 2	

### Data collection

**Table:**

Bruker SMART CCD area-detector diffractometer	1980 independent reflections
Radiation source: fine-focus sealed tube	1713 reflections with *I* > 2σ(*I*)
Graphite monochromator	*R*_int_ = 0.032
Detector resolution: 0 pixels mm^-1^	θ_max_ = 27.2°, θ_min_ = 2.2°
φ and ω scans	*h* = −6→5
Absorption correction: multi-scan (*SADABS*; Sheldrick, 1996)	*k* = −20→20
*T*_min_ = 0.960, *T*_max_ = 0.979	*l* = −15→14
8592 measured reflections	

### Refinement

**Table:**

Refinement on *F*^2^	Primary atom site location: structure-invariant direct methods
Least-squares matrix: full	Secondary atom site location: difference Fourier map
*R*[*F*^2^ > 2σ(*F*^2^)] = 0.039	Hydrogen site location: inferred from neighbouring sites
*wR*(*F*^2^) = 0.130	H-atom parameters constrained
*S* = 1.02	*w* = 1/[σ^2^(*F*_o_^2^) + (0.0876*P*)^2^ + 0.1852*P*] where *P* = (*F*_o_^2^ + 2*F*_c_^2^)/3
1980 reflections	(Δ/σ)_max_ < 0.001
119 parameters	Δρ_max_ = 0.34 e Å^−^^3^
0 restraints	Δρ_min_ = −0.34 e Å^−^^3^

### Special details

**Table:**

Geometry. All esds (except the esd in the dihedral angle between two l.s. planes) are estimated using the full covariance matrix. The cell esds are taken into account individually in the estimation of esds in distances, angles and torsion angles; correlations between esds in cell parameters are only used when they are defined by crystal symmetry. An approximate (isotropic) treatment of cell esds is used for estimating esds involving l.s. planes.
Refinement. Refinement of F^2^ against ALL reflections. The weighted R-factor wR and goodness of fit S are based on F^2^, conventional R-factors R are based on F, with F set to zero for negative F^2^. The threshold expression of F^2^ > 2sigma(F^2^) is used only for calculating R-factors(gt) etc. and is not relevant to the choice of reflections for refinement. R-factors based on F^2^ are statistically about twice as large as those based on F, and R- factors based on ALL data will be even larger.

### Fractional atomic coordinates and isotropic or equivalent isotropic displacement parameters (Å^2^)

**Table:**

	*x*	*y*	*z*	*U*_iso_*/*U*_eq_	
O1	−1.19587 (17)	0.35479 (5)	−0.09590 (7)	0.0219 (2)	
O2	−0.9567 (2)	0.37413 (6)	0.08774 (8)	0.0313 (3)	
O3	−0.30202 (17)	0.06258 (5)	−0.06554 (7)	0.0209 (2)	
C1	−1.5949 (3)	0.43801 (8)	−0.19241 (11)	0.0302 (3)	
H1A	−1.7199	0.4833	−0.1843	0.045*	
H1B	−1.7026	0.3869	−0.2106	0.045*	
H1C	−1.5046	0.4510	−0.2530	0.045*	
C2	−1.3723 (3)	0.42675 (7)	−0.08179 (11)	0.0234 (3)	
H2A	−1.2566	0.4772	−0.0643	0.028*	
H2B	−1.4617	0.4164	−0.0192	0.028*	
C3	−0.9916 (2)	0.33543 (7)	−0.00155 (10)	0.0199 (3)	
C4	−0.8119 (2)	0.26316 (7)	−0.01966 (10)	0.0188 (3)	
C5	−0.8593 (2)	0.21922 (7)	−0.12413 (10)	0.0199 (3)	
H5A	−1.0097	0.2346	−0.1851	0.024*	
C6	−0.6832 (2)	0.15314 (7)	−0.13669 (10)	0.0196 (3)	
H6A	−0.7145	0.1243	−0.2060	0.024*	
C7	−0.4580 (2)	0.12990 (7)	−0.04481 (10)	0.0174 (3)	
C8	−0.4063 (2)	0.17353 (7)	0.05940 (10)	0.0197 (3)	
H8A	−0.2549	0.1584	0.1201	0.024*	
C9	−0.5851 (2)	0.23998 (7)	0.07089 (10)	0.0197 (3)	
H9A	−0.5526	0.2693	0.1400	0.024*	
C10	−0.0671 (2)	0.03624 (7)	0.02485 (9)	0.0182 (3)	
H10A	−0.1317	0.0179	0.0912	0.022*	
H10B	0.0678	0.0820	0.0478	0.022*	

### Atomic displacement parameters (Å^2^)

**Table:**

	*U*^11^	*U*^22^	*U*^33^	*U*^12^	*U*^13^	*U*^23^
O1	0.0219 (4)	0.0201 (4)	0.0220 (4)	0.0073 (3)	0.0025 (3)	−0.0001 (3)
O2	0.0341 (5)	0.0299 (5)	0.0257 (5)	0.0111 (4)	−0.0005 (4)	−0.0075 (4)
O3	0.0185 (4)	0.0204 (4)	0.0212 (4)	0.0056 (3)	0.0002 (3)	−0.0036 (3)
C1	0.0313 (7)	0.0299 (7)	0.0275 (7)	0.0108 (5)	0.0034 (5)	0.0042 (5)
C2	0.0235 (6)	0.0183 (5)	0.0281 (6)	0.0065 (4)	0.0058 (5)	−0.0008 (4)
C3	0.0199 (6)	0.0182 (5)	0.0209 (6)	0.0009 (4)	0.0040 (4)	0.0011 (4)
C4	0.0188 (6)	0.0169 (5)	0.0210 (6)	0.0006 (4)	0.0053 (4)	0.0014 (4)
C5	0.0198 (6)	0.0192 (5)	0.0194 (6)	0.0013 (4)	0.0025 (4)	0.0023 (4)
C6	0.0217 (6)	0.0190 (5)	0.0176 (5)	−0.0001 (4)	0.0036 (4)	−0.0007 (4)
C7	0.0163 (5)	0.0154 (5)	0.0210 (6)	0.0002 (4)	0.0053 (4)	0.0007 (4)
C8	0.0176 (5)	0.0200 (5)	0.0200 (6)	0.0009 (4)	0.0019 (4)	0.0004 (4)
C9	0.0208 (6)	0.0186 (5)	0.0191 (6)	−0.0001 (4)	0.0039 (4)	−0.0022 (4)
C10	0.0158 (5)	0.0176 (5)	0.0200 (5)	0.0017 (4)	0.0021 (4)	−0.0008 (4)

### Geometric parameters (Å, °)

**Table:**

O1—C3	1.3446 (14)	C4—C5	1.4040 (16)
O1—C2	1.4604 (13)	C5—C6	1.3829 (15)
O2—C3	1.2109 (15)	C5—H5A	0.9300
O3—C7	1.3659 (13)	C6—C7	1.3977 (16)
O3—C10	1.4294 (13)	C6—H6A	0.9300
C1—C2	1.5027 (18)	C7—C8	1.3978 (16)
C1—H1A	0.9600	C8—C9	1.3925 (16)
C1—H1B	0.9600	C8—H8A	0.9300
C1—H1C	0.9600	C9—H9A	0.9300
C2—H2A	0.9700	C10—C10^i^	1.513 (2)
C2—H2B	0.9700	C10—H10A	0.9700
C3—C4	1.4874 (15)	C10—H10B	0.9700
C4—C9	1.3928 (16)		
			
C3—O1—C2	114.29 (9)	C6—C5—H5A	119.9
C7—O3—C10	117.60 (8)	C4—C5—H5A	119.9
C2—C1—H1A	109.5	C5—C6—C7	119.79 (10)
C2—C1—H1B	109.5	C5—C6—H6A	120.1
H1A—C1—H1B	109.5	C7—C6—H6A	120.1
C2—C1—H1C	109.5	O3—C7—C6	114.96 (10)
H1A—C1—H1C	109.5	O3—C7—C8	124.36 (10)
H1B—C1—H1C	109.5	C6—C7—C8	120.68 (10)
O1—C2—C1	107.71 (10)	C9—C8—C7	118.94 (11)
O1—C2—H2A	110.2	C9—C8—H8A	120.5
C1—C2—H2A	110.2	C7—C8—H8A	120.5
O1—C2—H2B	110.2	C8—C9—C4	120.89 (10)
C1—C2—H2B	110.2	C8—C9—H9A	119.6
H2A—C2—H2B	108.5	C4—C9—H9A	119.6
O2—C3—O1	123.03 (10)	O3—C10—C10^i^	105.17 (11)
O2—C3—C4	124.07 (11)	O3—C10—H10A	110.7
O1—C3—C4	112.89 (10)	C10^i^—C10—H10A	110.7
C9—C4—C5	119.45 (10)	O3—C10—H10B	110.7
C9—C4—C3	117.87 (10)	C10^i^—C10—H10B	110.7
C5—C4—C3	122.68 (11)	H10A—C10—H10B	108.8
C6—C5—C4	120.23 (11)		
			
C3—O1—C2—C1	−177.91 (10)	C10—O3—C7—C6	179.46 (9)
C2—O1—C3—O2	0.12 (16)	C10—O3—C7—C8	−1.26 (16)
C2—O1—C3—C4	−178.70 (9)	C5—C6—C7—O3	178.44 (9)
O2—C3—C4—C9	−1.66 (17)	C5—C6—C7—C8	−0.88 (16)
O1—C3—C4—C9	177.15 (9)	O3—C7—C8—C9	−178.42 (10)
O2—C3—C4—C5	179.57 (11)	C6—C7—C8—C9	0.83 (16)
O1—C3—C4—C5	−1.62 (16)	C7—C8—C9—C4	−0.16 (16)
C9—C4—C5—C6	0.41 (16)	C5—C4—C9—C8	−0.45 (16)
C3—C4—C5—C6	179.16 (10)	C3—C4—C9—C8	−179.26 (10)
C4—C5—C6—C7	0.25 (16)	C7—O3—C10—C10^i^	−178.04 (10)

Symmetry codes: (i) −*x*, −*y*, −*z*.

### Hydrogen-bond geometry (Å, °)

**Table:**

Cg is the centroid of the C4–C9 ring.

**Table:**

*D*—H···*A*	*D*—H	H···*A*	*D*···*A*	*D*—H···*A*
C6—H6A···O2^ii^	0.93	2.47	3.2784 (16)	146.
C10—H10B···Cg^iii^	0.97	2.65	3.741 (2)	143

Symmetry codes: (ii) *x*, −*y*+1/2, *z*−1/2; (iii) *x*+1, *y*, *z*.

## References

[bb1] Allen, F. H., Kennard, O., Watson, D. G., Brammer, L., Orpen, A. G. & Taylor, R. (1987). *J. Chem. Soc. Perkin Trans 2*, pp. S1–19.

[bb2] Bruker (2001). *SMART* Bruker AXS Inc., Madison, Wisconsin, USA.

[bb3] Bruker (2002). *SAINT* Bruker AXS Inc., Madison, Wisconsin, USA.

[bb4] Chen, X. & Liu, G. (2002). *Chem. Eur. J.* **8**, 4811–4817. 10.1002/1521-3765(20021018)8:20<4811::AID-CHEM4811>3.0.CO;2-R 12561122

[bb5] Hou, L.-M. & Kan, Y.-H. (2007). *Acta Cryst.* E**63**, o2157–o2158.

[bb6] Ma, Z. & Liu, S.-X. (2002). *Chin. J. Struct. Chem* **21**, 533–537.

[bb16] Ma, Z. & Cao, Y. (2011). *Acta Cryst.* E**67**, o1503. 10.1107/S1600536811018150 PMC3120494 21754870

[bb7] Sheldrick, G. M. (1996). *SADABS* University of Göttingen, Germany.

[bb8] Sheldrick, G. M. (2008). *Acta Cryst.* A**64**, 112–122. 10.1107/S0108767307043930 18156677

[bb9] Tashiro, K., Hou, J., Kobayashi, M. & Inoue, T. (1990). *J. Am. Chem. Soc.* **112**, 8273–8279.

[bb10] Zhang, L.-P., Jia, Z.-F., Wei, G.-H. & Liu, Y.-Y. (2007). *Acta Cryst.* E**63**, o4674.

